# *CFH* 184G as a genetic risk marker for anterior uveitis in Chinese females

**Published:** 2011-10-13

**Authors:** Ming-ming Yang, Timothy Y.Y. Lai, Pancy O.S. Tam, Sylvia W.Y. Chiang, Carmen K.M. Chan, Fiona O.J. Luk, Tsz Kin Ng, Chi-Pui Pang

**Affiliations:** Department of Ophthalmology and Visual Sciences, The Chinese University of Hong Kong, Hong Kong, China

## Abstract

**Objective:**

To investigate the association of three single nucleotide polymorphisms (SNPs) in the complement factor H (*CFH*), *KIAA1109*, and interleukin-27 (*IL-27*) genes in patients with anterior uveitis (AU).

**Methods:**

A case-control study was performed in 98 Chinese AU patients and 308 healthy controls. Three SNPs including *CFH*-rs800292, *KIAA1109*-rs4505848, and *IL27*-rs4788084 were detected using TaqMan SNP Genotyping Assays. Analyses were also stratified according to gender, clinical features and human leukocyte antigen (HLA)-B27 status of the patients.

**Results:**

No significant association was found between all three SNPs and AU. However, when stratified by gender, there were significant increases in the frequency of the *CFH*-rs800292 184G allele and GG homozygosity in female patients compared with control subjects (p=0.003 and p=0.009, respectively). Similar association was not detected in males. No significant association was found between AU and *KIAA1109*-rs4505848 or *IL27*-rs4788084 even stratified by gender. There was no significant difference in genotypes of AU patients stratified by various clinical features. Subgroup analyses showed that all three SNPs (rs800292, rs4505848, and rs4788084) were not associated with AU in HLA-B27–positive patients, neither in HLA-B27–negative patients.

**Conclusions:**

Our results showed an association between AU and *CFH* polymorphism in Chinese female patients but not in males, indicating gender-specific genetic differences in *CFH*. Gender should be considered in genetic studies of anterior uveitis even extending to other immunologic diseases.

## Introduction

Uveitis is an intraocular inflammatory disease involving the uveal tract and can be anatomically classified as anterior, intermediate, posterior, and panuveitis [1]. Anterior uveitis (AU) is the most common form, accounting for 50% to 92% of cases in most Western countries and 28% to 50% in Asian countries [2]. AU involves the anterior part of the uveal tract and can be idiopathic or associated with other immunologic disorders such as ankylosing spondylitis (AS) and rheumatoid arthritis (RA). Although the exact pathogenesis of AU is unclear, clinical and animal studies have demonstrated that the inflammation in AU is regulated by various endogenous immunological factors. In addition, an important conceptual consideration is that the disease can manifest in individuals with genetic predisposition coupled with environmental trigger [3,4]. AU is closely associated with human leukocyte antigen (HLA)-B27, the major histocompatibility complex (MHC) type I gene [5,6]. Gene polymorphisms of various non-MHC genes such as cytokines and antioxidant enzyme genes have also been implicated to play important roles in the pathogenesis of AU [7-9].

Interleukins are potent inflammatory mediators produced by white blood cells and play important roles in the development of uveitis [10-16]. Studies have shown that the levels of interleukin 2 (IL-2), interleukin 21 (IL-21) and their receptors were upregulated in both experimental autoimmune uveitis (EAU) animals and in uveitis patients [10-13]. Recently, several studies have revealed that IL-27 expression was upregulated during uveitis and retinal cells could suppress uveitis through production of IL-27 [15,16]. Several single nucleotide polymorphisms (SNPs) in interleukin genes have been found to be associated with various types of uveitis but only a few of them were found to have a strong association [17-19]. In recent years, based on genome-wide association study (GWAS), several candidate SNPs were successively found to be associated with a range of immune-mediated diseases, such as Behçet’s disease, type 1 diabetes mellitus, RA, celiac disease, and Graves’ disease [20-24]. Some of these genetic loci were replicated reciprocally in various diseases, implying that these loci might be the general risk factors for multiple autoimmune diseases [25-27].

The complement system is another important pathway of innate immunity. The system can be divided into the classic, lectin and alternative pathways and plays an important role in many immunological diseases including uveitis. Various studies have demonstrated that the activation of complement system is critical for the development of experimental autoimmune anterior uveitis (EAAU) and the depletion of the host’s complement system could result in complete inhibition of EAAU [28,29]. Under normal conditions, complement system is continuously active at a low level and is tightly regulated by intraocular complement regulatory proteins (CRegs) such as complement factor H (CFH), decay-accelerating factor and S-protein. Studies have shown that suppression of CRegs can exacerbate EAAU due to unregulated complement activation [30,31]. CFH acts as a key regulator in the complement alternative pathway and is involved in the pathogenesis of many immunological diseases [32-34]. Recent studies have suggested that variants in the *CFH* gene, as well as in the genes of other CFH-related proteins might be involved in many immune-mediated diseases [35-37]. We hypothesized that rs4505848 within the *KIAA1109*/Testis nuclear RNA-binding protein (*Tenr*)/*IL2*/*IL21* gene cluster, rs4788084 in *IL-27*, and rs800292 in *CFH* might be involved in the pathogenesis of AU. The purpose of our study is to determine the association of these immune-associated SNPs in Chinese patients with AU.

## Methods

### Study design and subjects

Subjects were recruited from the Hong Kong Eye Hospital. The study protocol was approved by an institutional review board and all the procedures were conducted according to the tenets of the Declaration of Helsinki. Informed consent was obtained from all study subjects after explanation of the nature of the study.

All patients underwent detailed ocular examination including visual acuity, intraocular pressure, slit lamp, and dilated fundus examinations. Clinical details were also collected from the case notes including age, sex, medical history such as systemic illness, age at initial presentation, laterality, pattern of anterior uveitis (acute, recurrent or chronic), clinical features, and complications of AU. The definition of uveitis was based on the Standardization Uveitis Nomenclature (SUN) classification [38]. Acute AU was defined as AU resolving completely within 3 months, chronic AU as AU not fully resolved within 3 months, and recurrent AU as the development of AU more than once. Patients with any of the following situations were excluded from the study (1) AU secondary to ocular or systemic infections; (2) AU secondary to specific syndromes (e.g., Posner-Schlossman’s syndrome, Fuchs’ uveitis, Vogt–Koyanagi–Harada (VKH) or Behçet’s disease); (3) patients who were unable to cooperate during ocular examination and with chronic uveitis at the onset of the study. Three hundred and eight subjects aged 50 years older with no ophthalmic eye disease except senile cataract were included as control subjects.

### DNA extraction and genotyping

Venous blood was obtained from each subject and genomic DNA was extracted with a DNA extraction kit (QIAamp; Qiagen, Hilden, Germany) according to the manufacturer’s instructions. *CFH*-rs800292, *KIAA1109*-rs4505848, and *IL27*-rs4788084 SNPs were genotyped by TaqMan allelic discrimination assay (TaqMan; Applied Biosystems [ABI], Foster City, CA) according to the manufacturer’s instructions. All PCR amplifications were performed with the following thermal cycling conditions: 95 °C for 10 min followed by 40 cycles of 92 °C for 15 s, and 62 °C for 1.5 min (rs4505848 and rs4788084); and 60 °C for 1 min (rs800292), respectively. The HLA-B27 allele was detected by nested PCR as described by Konno et al. [39]. The B locus of HLA was first amplified, the B27 allele was then amplified from the diluted PCR product of the B locus by using sequence-specific primers. The B27 allele was further detected and confirmed as described previously [39]. All PCR reactions were performed with Taq polymerase (HotStarTaq Plus; Qiagen) in an automated thermal cycler (model 9700; ABI). Pre- and post-PCR plate readings were performed on a sequence detection system (Prism 7000; ABI), and the allele types were confirmed by the system software (Prism 7000 SDS software version 1.1; ABI).

### Statistical analysis

Hardy–Weinberg equilibrium (HWE) for genotype frequencies of the SNPs in the control group were tested by *χ^2^* test. The genotype frequencies for each polymorphism were determined by direct counting and allele frequencies were calculated. Allelic and genotypic frequencies between patients with AU and controls were compared by *χ^2^* test. Analyses were also performed by stratifying AU patients based on gender and HLA-B27 status and compared with control subjects. Odds ratios (OR) and 95% confidence intervals (CI) were calculated. Bonferroni correction was applied to adjust for the number of comparisons performed based on the total number of loci and thus a p value of 0.017 was considered as statistically significant.

## Results

### Patients demographics

Ninety-eight patients with AU were recruited, including 45 (45.9%) males and 53 (54.1%) females. The mean±SD age of the patients was 49.7±16.0 years (range, 11–87 years). Forty-seven (48.0%) patients had unilateral uveitis, and 51 (52.0%) had bilateral involvement. Systemic diseases associated with the patients included AS (n=19, 19.4%); psoriasis (n=1, 1.0%); systemic lupus erythematosus (n=1, 1.0%); ulcerative colitis (n=1, 1.0%), and interstitial nephritis (n=1, 1.0%). Ninety-two (93.9%) patients had acute AU only, of which 59 (60.2%) had recurrent episodes of AU, and 6 (6.1%) developed chronic AU after acute episodes of AU.

### Associations between SNPs and AU

All genotype frequencies of the 3 SNPs in the control subjects conformed to the Hardy–Weinberg equilibrium. There was no significant difference in allelic and genotypic frequencies for the 3 SNPs (*CFH*-rs800292, *KIAA1109*-rs4505848, and *IL27*-rs4788084) in AU patients compared with controls. There was a trend toward higher 184G allele frequency for rs800292 in AU patients compared with controls but the difference did not reach the level of statistical significance (31.6% versus 39.1%, p=0.059; Table 1).

**Table 1:** Comparison of genotype and allele frequencies of rs800292, rs4505848, and rs4788084 polymorphisms in patients with AU and control subjects.

**Polymorphism**	**Anterior uveitis (n=98)**	**Controls (n=308)**	**p-value**	**Odds ratio**
rs800292** (*CFH* 184G/A)**				
**Genotype**				
AA	10 (10.2)	48 (15.6)	0.17§	
AG	42 (42.9)	145 (47.1)	0.091*	
GG	46 (46.9)	115 (37.3)	0.19‡	
**Allele**				
A	62 (31.6)	241 (39.1)	0.059§	1.39 (0.99–1.96)
G	134 (68.4)	375 (60.9)		
rs4505848** (*KIAA1109* G/A)**				
**Genotype**				
GG	12 (12.2)	50 (16.2)	0.42§	
AG	57 (58.2)	157 (51.0)		
AA	29 (29.6)	101 (32.8)		
**Allele**				
G	81 (41.3)	257 (41.7)	0.92§	
A	115 (58.7)	359 (58.3)		
rs4788084** (*IL27* T/C)**				
**Genotype**				
TT	9 (9.2)	21 (6.8)	0.74§	
CT	39 (39.8)	126 (40.9)		
CC	50 (51.0)	161 (52.3)		
**Allele**				
T	57 (29.1)	168 (27.3)	0.62§	
C	139 (70.9)	448 (72.7)		

Data are the number of subjects (% of the total group). §χ^2^ test. *p-value for dominant model. ‡p-value for recessive model.

### Associations between SNPs and AU stratified by gender and clinical features

When the analyses were stratified on the basis of gender, there was a significant increase in the frequency of 184G allele and GG homozygosity for the *CFH*-rs800292 SNP in female AU patients compared with control female subjects (p=0.003, OR=2.10 and p=0.009, OR=2.28, respectively). However, similar difference was not observed in male patients. For the *KIAA1109*-rs4505848 and *IL27*-rs4788084, there was no significant difference in both male and female AU patients compared with controls (Table 2 and Table 3). There was no significant difference in allelic and genotypic frequencies in AU patients stratified by recurrence and laterality (Table 4 and Table 5).

**Table 2:** Comparison of genotype and allele frequencies of rs800292, rs4505848, and rs4788084 polymorphisms in female patients with AU and female control subjects.

**Polymorphism**	**Female AU patients (n=53)**	**Female controls (n=183)**	**p-value**	**Odds ratio (95% confidence interval)**
rs800292** (*CFH* 184G/A)**				
**Genotype**				
AA	3 (5.7)	31 (16.9)	0.015§	
AG	19 (35.8)	82 (44.8)	0.009*	2.28 (1.22–4.24)
GG	31 (58.5)	70 (38.3)	0.039‡	3.40 (1.00–11.6)
**Allele**				
A	25 (23.6)	144 (39.3)	0.003§	2.10 (1.28–3.45)
G	81 (76.4)	222 (60.7)		
rs4505848** (*KIAA1109* G/A)**				
**Genotype**				
GG	7 (13.2)	27 (14.8)	0.54§	
AG	32 (60.4)	95 (51.9)		
AA	14 (26.4)	61 (33.3)		
**Allele**				
G	46 (43.4)	149 (40.7)	0.62§	
A	60 (56.6)	217 (59.3)		
rs4788084** (*IL27* T/C)**				
**Genotype**				
TT	8 (15.1)	12 (6.6)	0.13§	
CT	19 (35.8)	78 (42.6)		
CC	26 (49.1)	93 (50.8)		
**Allele**				
T	35 (33.0)	102 (27.9)	0.30§	
C	71 (67.0)	264 (72.1)		

Data are the number of subjects (% of the total group). §χ^2^ test. *p-value for dominant model. ‡p-value for recessive model.

**Table 3:** Comparison of genotype and allele frequencies of rs800292, rs4505848, and rs4788084 polymorphisms in male patients with AU and male control subjects.

**Polymorphism**	**Male AU patients (n=45)**	**Male controls (n=125)**	**p-value**
rs800292** (*CFH* 184G/A)**			
**Genotype**			
AA	7 (15.6)	17 (13.6)	0.92§
AG	23 (51.1)	63 (50.4)	0.75*
GG	15 (33.3)	45 (36.0)	0.75‡
**Allele**			
A	37 (41.1)	97 (38.8)	0.70§
G	53 (58.9)	153 (61.2)	
rs4505848** (*KIAA1109* G/A)**			
**Genotype**			
GG	5 (11.1)	23 (18.4)	0.52§
AG	25 (55.6)	62 (49.6)	
AA	15 (33.3)	40 (32.0)	
**Allele**			
G	35 (38.9)	108 (43.2)	0.48§
A	55 (61.1)	142 (56.8)	
rs4788084** (*IL27* T/C)**			
**Genotype**			
TT	1 (2.2)	9 (7.2)	0.43§
CT	20 (44.4)	48 (38.4)	
CC	24 (53.3)	68 (54.4)	
**Allele**			
T	22 (24.4)	66 (26.4)	0.72§
C	68 (75.6)	184 (73.6)	

Data are the number of subjects (% of the total group). §χ^2^ test. *p-value for dominant model. ‡p-value for recessive model.

**Table 4:** Comparison of genotype and allele frequencies of rs800292, rs4505848, and rs4788084 polymorphisms in AU patients stratified by recurrence status.

**Polymorphism**	**Recurrent (n=59)**	**Non-recurrent (n=39)**	**p-value**
rs800292** (*CFH* 184G/A)**			
**Genotype**			
AA	5 (8.5)	5 (12.8)	0.38§
AG	23 (39.0)	19 (48.7)	
GG	31 (52.5)	15 (38.5)	
**Allele**			
A	33 (28.0)	29 (37.2)	0.18§
G	85 (72.0)	49 (62.8)	
rs4505848** (*KIAA1109* G/A)**			
**Genotype**			
GG	7 (11.9)	5 (12.8)	0.51§
AG	32 (54.2)	25 (64.1)	
AA	20 (33.9)	9 (23.1)	
**Allele**			
G	46 (39.0)	35 (44.9)	0.41§
A	72 (61.0)	43 (55.1)	
rs4788084** (*IL27* T/C)**			
**Genotype**			
TT	7 (11.9)	2 (5.1)	0.50§
CT	22 (37.3)	17 (43.6)	
CC	30 (50.8)	20 (51.3)	
**Allele**			
T	36 (30.5)	21 (26.9)	0.59§
C	82 (69.5)	57 (73.1)	

Data are the number of subjects (% of the total group). §χ^2^ test.

**Table 5:** Comparison of genotype and allele frequencies of rs800292, rs4505848, and rs4788084 polymorphisms stratified by laterality status.

**Polymorphism**	**Bilateral AU (n=51)**	**Unilateral AU (n=47)**	**p-value**
rs800292** (*CFH* 184G/A)**			
**Genotype**			
AA	4 (7.8)	6 (12.8)	0.70§
AG	23 (45.1)	19 (40.4)	
GG	24 (47.1)	22 (46.8)	
**Allele**			
A	31 (30.4)	31 (33.0)	0.70§
G	71 (69.6)	63 (67.0)	
rs4505848** (*KIAA1109* G/A)**			
**Genotype**			
GG	7 (13.7)	5 (10.6)	0.55§
AG	27 (52.9)	30 (63.8)	
AA	17 (33.3)	12 (25.5)	
**Allele**			
G	41 (40.2)	40 (42.6)	0.74§
A	61 (59.8)	54 (57.4)	
rs4788084** (*IL27* T/C)**			
**Genotype**			
TT	4 (7.8)	5 (10.6)	0.53§
CT	23 (45.1)	16 (34.0)	
CC	24 (47.1)	26 (55.3)	
**Allele**			
T	31 (30.4)	26 (27.7)	0.67§
C	71 (69.6)	68 (72.3)	

Data are the number of subjects (% of the total group). §χ^2^ test for 2×3.

### Association of SNPs genotypes and allele frequencies stratified by HLA-B27 status

Forty-two (42.9%) of the 98 patients with AU were HLA-B27-positive and fifty-six (57.1%) were HLA-B27-negative. When analyzed on the basis of HLA-B27 status, there was no significant difference in allelic and genotypic frequencies for the 3 SNPs (*CFH*-rs800292, *KIAA1109*-rs4505848, and *IL27*-rs4788084) between either HLA-B27-positive or HLA-B27-negative patients compared with control subjects (Table 6).

**Table 6:** Comparison of genotype and allele frequencies of rs800292, rs4505848, and rs4788084 polymorphisms in patients with AU versus control subjects stratified by HLA-B27 status.

**Polymorphism**	**HLA-B27-positive anterior uveitis (n=42)**	**HLA-B27-negative anterior uveitis (n=56)**	**Controls (n=308)**	**p-value†**	**p-value‡**
rs800292** (*CFH* 184G/A)**					
**Genotype**					
AA	3 (7.1)	7 (12.5)	48 (15.6)	0.30§	0.31§
AG	20 (47.6)	22 (39.3)	145 (47.1)		
GG	19 (45.2)	27 (48.2)	115 (37.3)		
**Allele**					
A	26 (31.0)	36 (32.1)	241 (39.1)	0.15§	0.16§
G	58 (69.0)	76 (67.9)	375 (60.9)		
rs4505848** (*KIAA1109* G/A)**					
**Genotype**					
GG	4 (9.5)	8 (14.3)	50 (16.2)	0.24§	0.91§
AG	27 (64.3)	30 (53.6)	157 (51.0)		
AA	11 (26.2)	18 (32.1)	101 (32.8)		
**Allele**					
G	35 (41.6)	46 (41.1)	257 (41.7)	0.99§	0.90§
A	49 (58.4)	66 (58.9)	359 (58.3)		
rs4788084** (*IL27* T/C)**					
**Genotype**					
TT	4 (9.5)	5 (8.9)	21 (6.8)	0.75§	0.80§
CT	18 (42.9)	21 (37.5)	126 (40.9)		
CC	20 (47.6)	30 (53.6)	161 (52.3)		
**Allele**					
T	26 (31.0)	31 (27.7)	168 (27.3)	0.48§	0.93§
C	58 (69.0)	81 (72.3)	448 (72.7)		

Data are the number of subjects (% of the total group). §χ^2^ test. †p-value for HLA-B27-positive. Patients versus controls. ‡p-value for HLA-B27-negative patients versus controls.

## Discussion

In our study, we investigated the association of three immune-associated SNPs in the *CFH*, *KIAA1109*, and *IL27* in Chinese patients with AU. Our results demonstrated there was a trend toward higher 184G allele frequency in the *CFH*-rs800292 SNP among AU patients compared with controls. Although the difference failed to reach the level of statistical significance (p=0.059), the findings suggested that *CFH* might be associated with the susceptibility of developing AU. Since genetic and environmental factors might be associated with some gender-specific differences in the severity and type of immunological diseases, we therefore further evaluated the association stratified by gender. Our results showed a significant increase in the frequency of 184G allele and GG homozygousity in female AU patients compared with control subjects. To our knowledge, the associations between genetic variants in *CFH* and AU and the gender differences have not been described previously. Activated complement is a “double-edged sword” which might cause self-tissue damage especially in a sensitive organs like the eyes and they must be carefully regulated by CRegs such as CFH [40]. *CFH* is located in the long arm of chromosome 1 (1q32), which is a major soluble inhibitor of the alternative pathway in controlling complement activation [30]. Furthermore, in vivo studies have revealed that human retinal pigment epithelial (RPE) cells can synthesize and express CFH, which upregulates various CRegs including CFH to suppress the development of EAU [31,41,42].

In our previous study, we found SNP rs800292 in *CFH* was associated with age-related macular degeneration (AMD) in Chinese patients [43]. In addition, *CFH* has also been found to be associated with other immune-mediated diseases such as multifocal choroiditis, hemolytic-uremic syndrome (HUS) and glomerulonephritis, where some risk loci overlap with those of AMD [35,36]. In this study, the SNP rs800292 was found to be associated with AU in female patients. The change of G184A nucleotide of rs800292 in the *CFH* gene results in the synthesis of Isoleucine instead of Valine at codon 62. This might lead to structural changes affecting the ability of complement component 3b (C3b) binding and reducing the activation of the alternative pathway C3-convertase (C3bBb). This subsequently causes excessive activation of the complement system to induce immunologic disorder and the gender bias may be due to different pathway of CFH in AU. The exact mechanism is still unclear and further studies will be needed to investigate the functional interaction of CFH and AU and the difference in gender-susceptibility in AU.

SNP rs4505848 is in the region encompassing *KIAA1109/Tenr/IL2/IL21* which is located on the chromosome 4q27. This 480 kB block of linkage disequilibrium includes *IL2* and *IL21*, which are both functional candidate loci for autoimmune diseases. As mentioned previously, levels of IL2, IL21, and their receptors were significantly increased during uveitis in patients and animal models and they may participate in the regulation of T-cell responses [11-13]. Current evidences indicated that both T helper 1 (Th1) and T helper 17 (Th17) effector cells can independently induce uveitis in animal models [44,45]. Functional studies have revealed that Th17 cells contribute to uveitis through expanded IL-2, meanwhile IL-21 was highly expressed and promoted the differentiation of Th17 cells both in vitro and in vivo studies [13,15]. IL27 is a recently discovered cytokine belonging to the IL6/IL12 families, which consists of EBI3 and p28 subunits. Studies have shown that IL27 was constitutively expressed in retinal ganglion and photoreceptor cells in EAU and IL27 could promote Th1 but inhibit Th17 cells differentiation, which lead to a mutual antagonism between the two pathways [15].

Taken together, these studies indicate that IL2, IL21, and IL27 play important roles in the development of uveitis, predicting an association of these two SNPs (rs4505848 and rs4788084) with AU. However in our study, no such association was found even stratified by gender. This might be due to ethnic differences in susceptibility to AU; the small number of subjects; the wide variety of AU syndromes; the complex regulation mechanism in inflammatory activity, as well as the candidate polymorphisms chosen among these plausible genes. Therefore, further investigations are needed in more subtypes of uveitis and other ethnic groups. In addition, considering HLA-B27 is the strongest association with AU and the interaction with these immune-associated genes, further analyses were performed in these AU patients stratified by HLA-B27 status. We found all the 3 SNPs were not associated with AU regardless of HLA-B27 positivity. The exact reason is unclear but it may account for the weaker association of these SNPs with AU, or the influence of these genes on AU independent on HLA-B27.

In conclusion, we found the association of *CFH* 184G with AU in Chinese female patients. The genetic association between the complement system and AU was identified with gender susceptibility. Further studies to replicate the candidate SNPs in others ethnic groups and determine the biologic roles of these polymorphisms in immune mechanisms involved in uveitis are worthwhile.
